# MTX-PEG-modified CG/DMMA polymeric micelles for targeted delivery of doxorubicin to induce synergistic autophagic death against triple-negative breast cancer

**DOI:** 10.1186/s13058-022-01599-9

**Published:** 2023-01-12

**Authors:** Zhiwen Cao, Rui Liu, Yang Li, Xinyi Luo, Zhenglai Hua, Xiangpeng Wang, Zeyu Xue, Zhengjia Zhang, Cheng Lu, Aiping Lu, Yuanyan Liu

**Affiliations:** 1 School of Chinese Materia Medica, Beijing University of Chinese Medicine, Beijing, 100029 China; 2 Institute of Basic Research in Clinical Medicine, China Academy of Chinese Medical Sciences, Beijing, 100700 China; 3 School of Chinese Medicine, Hong Kong Baptist University, Kowloon,, Hongkong China

**Keywords:** Triple-negative breast cancer, Autophagy, Doxorubicin, Chitosan, Micelles

## Abstract

The chemotherapy of triple-negative breast cancer based on doxorubicin (DOX) regimens suffers from great challenges on toxicity and autophagy raised off-target. In this study, a conjugate methotrexate-polyethylene glycol (shorten as MTX-PEG)-modified CG/DMMA polymeric micelles were prepared to endue DOX tumor selectivity and synergistic autophagic flux interference to reduce systematic toxicity and to improve anti-tumor capacity. The micelles could effectively promote the accumulation of autophagosomes in tumor cells and interfere with the degradation process of autophagic flux, collectively inducing autophagic death of tumor cells. In vivo and in vitro experiments showed that the micelles could exert improved anti-tumor effect and specificity, as well as reduced accumulation and damage of chemotherapeutic drugs in normal organs. The potential mechanism of synergistic autophagic death exerted by the synthesized micelles in MDA-MB-231 cells has been performed by autophagic flux-related pathway.

**Supplementary Information:**

The online version contains supplementary material available at 10.1186/s13058-022-01599-9.

## Introduction

Breast cancer is the major cause of morbidity and mortality among malignant tumors in women [1–3]. It is worth noting that triple-negative breast cancer (TNBC) accounts for approximately 10–15% of all breast cancers and its malignant is characterized by the worst prognosis, increased recurrence rates regardless of the stage of the disease and resistance to chemotherapy [4, 5]. To some extent, the refractoriness and chemotherapeutic failure of TNBC may attribute to the higher basal levels of autophagy with self-healing in tumor cells than that in normal cells [1, 6–8]. Meanwhile, in the progression of TNBC, autophagy contributes to tumor survival by providing nutrition, regulating oxidative stress and promoting drug resistance [6, 9]. As an evolutionary conserved cellular process, autophagy is aroused by the recognition of disposable or potentially harmful cytoplasmic entities and is culminated in the lysosomal degradation [10, 11]. Basal autophagy generally promotes cell survival as a protective process. Paradoxically, excessive or sustained autophagy leads to extreme levels of autophagic flux and subsequently excessive consumption of organelles and cytoplasmic contents, then promoting cell death. In this sense, autophagy is considered to be a type II programming cell death or autophagic death [12–14]. Given that, we propose a hypothesis that ingeniously designed synergistic therapy might emerge as an efficient strategy for interfering with the multistep process of autophagy to induce autophagic death on tumor cell itself and escaping the degradation on drugs.

Currently, new targeted therapies on TNBC have been developed, including immune checkpoint inhibitors, androgen receptor or poly ADP-ribose polymerase, but treatment options were still limited and cytotoxic chemotherapy remained the dominant treatment [7, 15]. Doxorubicin (DOX) is a conventional chemotherapeutic drug for TNBC, whose mechanism is mainly to inhibit tumor proliferation by interfering with mRNA and DNA synthesis of tumor cells [16, 17]. Although the anti-tumor activity of DOX is quite robust, its clinical applications are greatly limited due to its irreversible tissue toxicity and drug resistance aroused off-target [18–20]. In the present study, nano-scaled polymeric micelles were designed for targeted delivery of DOX to reduce toxicity and interference on autophagic process [10, 21, 22].

Nano-engineered delivery systems have received extensive attention due to their promotion of tumor tissue accumulation and retention through enhanced permeability and retention (EPR) effects and receptor-mediated ligand targeting to tumor cells, whereas the endo/lysosomal barrier is a major challenge for delivering and releasing drugs into cytoplasm [19, 23]. Chitosan (CG), a natural polysaccharide, is widely developed in drug delivery system owning to its biocompatibility, biodegradability, cell adhesion and tumor inhibition [23–25]. In addition, 2,3-dimethylmaleic anhydride (DMMA) appropriately modified to CG could capture protons from the outside of lysosome, and the concomitantly entered chloride ions and water into lysosome might induce potential rupture (proton sponge effect).

Based on the above, a conjugate methotrexate-polyethylene glycol (MTX-PEG)-modified CG/DMMA polymeric micelles were designed and synthesized. Specifically, CG was connected by DMMA to form a polymer with negative charge, and DOX was loaded into the delivery system by electrostatic interactions [24, 26]. To improve the stability and targeting of the system, in this design, the surface of the micelles was modified with polyethylene glycol (PEG) connected to methotrexate (MTX) at one end. PEG can shield protein adsorption and avoid becoming a protein crown in the blood circulation [27, 28]. MTX can specifically bind to the folic acid receptor overexpressed on the surface of tumor cells to promote tumor targeting and reduce the toxicity of DOX [29]. The effect of the synthesized micelles on autophagy and its potential mechanism was detected by MDA-MB-231 cells in vitro, and its anti-tumor effect and selectivity were measured in tumor-bearing mice. On the one hand, tumor-targeting ability can effectively promote DOX aggregation in tumor tissue to exert therapeutic effect; on the other hand, DOX and the micelles-induced autophagosomes lead to autophagic death of tumor cells due to lysosomal damage and autophagic flux blockage [18, 30]. All the results showed that the CG/DMMA polymeric micelles based on MTX-PEG modification could effectively improve the targeting and therapeutic effect of DOX, while the autophagic flux blockage effect of the micelles could also exert synergistic anti-tumor effect.

## Materials and methods

### Reagents and materials

Doxorubicin (DOX) and methotrexate (MTX) were obtained from Beijing Solarbio Science & Technology Co., Ltd. 2,3-Dimethylmaleic anhydride (DMMA) was sourced from Shanghai Yuanye Bio-Technology Co., Ltd. MTX-PEG was obtained from Yarebio. Chitosan (CG), 1-ethyl-3(3-dimethylaminopropyl) carbodiimide (EDC), N-hydroxysuccinimide (NHS), 4′-6-diamidino-2-phenylindole dihydrochloride (DAPI) and dimethyl sulfoxide (DMSO) were obtained from Sigma-Aldrich (Shanghai) Trading Co. Ltd. Cell Counting Kit-8 (CCK-8) was sourced from Dojindo Laboratories. Fetal bovine serum (FBS) was sourced from Gibco Laboratories. 1% antibiotics (streptomycin 100 mg/mL and penicillin 100 U/mL), DMEM-H/F-12 medium and DMEM/HIGH Glucose medium were obtained from HyClone Laboratories. Antibodies for Beclin-1 (D40C5), LC3A/B (D3U4C), SQSTM1/p62 (D5L7G) and GAPDH (D16H11) were sourced from Cell Signaling Technology.

### Preparation of CG-MTX-PEG

CG-MTX-PEG was synthesized through the catalysis of EDC and NHS according to previous reports. In brief, CG (120 mg), HOOC-MTX-PEG (76 mg), NHS (33.2 mg) and EDC (55.2 mg) were stirred in 12 mL of deionized water at room temperature. After 48 h, the reaction solution was dialyzed in deionized water for 3 days to separate excess catalyst (MWCO = 3500 Da) and then lyophilized to obtain a yellow flocculent product CG-MTX-PEG. The sample was stored at − 20 °C.

### Preparation of CG/DMMA-MTX-PEG (CDPM)

The amidation reaction of CG-MTX-PEG and DMMA was completed under triethylamine (TEA) catalysis according to the previous report [31]. In short, 50 mg of CG-MTX-PEG and 40 mg of DMMA were dissolved in 10 mL of DMSO, and then, 50 μL of TEA was added, and the reaction was stirred for 24 h at room temperature. Subsequently, the reaction solution was transferred into a dialysis tube (MWCO = 3500 Da) and dialyzed in deionized water with pH (8–9) adjusted by NaOH solution to remove catalyst and excess DMMA. The solution was lyophilized to obtain the final product CG/DMMA-MTX-PEG (CDPM), and the sample was stored at − 20 °C.

### Preparation of DOX-loaded micelles

In brief, CDPM and DOX were dissolved in 30 mL DMSO under dark conditions. After vigorous stirring for 1 h, the mixture was slowly dropped into 10 mL deionized water and stirred. Finally, the mixture solution was transferred to a dialysis tube (MWCO = 3500 Da) and dialyzed to form micelles.

### Characterizations

The infrared spectra of different samples were measured by Fourier infrared spectroscopy (FT-IR) with the KBr method over the wavenumber range of 4000–400 cm^−1^. The Zeta potential and size distribution of the micelles were obtained by Zetasizer. The morphology of the micelles was observed by a transmission electron microscope (TEM).

### Protein adsorption

Bovine serum albumin (BSA) was used as plasma protein model to evaluate the protein adsorption effect of the micelles in this experiment. The sample solutions were separately incubated with BSA solution in ddH_2_O, and the final micelle and protein concentrations were 0.5 and 0.3 mg/mL, respectively. The mixture solutions were incubated at 37 °C for 2 h and then centrifuged for 10 min at 12,000 rpm to precipitate the protein adsorption aggregates completely. The supernatant was separated, and the concentration of BSA was determined and calculated according to the BCA protein assay kit.

### In vitro drug release

The method of dialysis was used to study in vitro release behavior of drugs. The PBS solution with different pH value was used to simulate the microenvironment of tumor and normal tissue. Briefly, the micelles were dissolved in 5 mL of PBS solution and transferred to dialysis tubes (MWCO = 3500 Da). The dialysis tube was immersed in 25 mL PBS buffer, and the entire dialysis system was maintained at 37 °C, 120 rpm. At desired time intervals, 2 mL of dialysate was taken out and then replaced with the same volume of PBS solution. In order to evaluate the release of MTX, 1 mg/mL protease was added to simulate the lysosomal environment. The concentration of DOX was detected by fluorescence intensity at 590 nm (*λ*_ex_: 490 nm), and the concentration of MTX was obtained by measuring absorbance (305 nm).

### Cell culture

MDA-MB-231 cells were purchased from BeNa Culture Collection (BNCC) and cultured in CM1-1 medium (90% DMEM/HIGH containing 10% (V/V) FBS) in an atmosphere of 5% CO_2_ (V/V) at 37 °C. HK-2 cells were purchased from American Type Culture Collection (ATCC) and cultured in CM9-1 medium (90% DMEMH/F-12 containing 10% (V/V) FBS) in an atmosphere of 5% CO_2_ (V/V) at 37 °C.

### Cellular uptake

The cellular uptake behavior of the micelles was verified by confocal laser scanning microscopy (CLSM). MDA-MB-231 cells (1 × 10^4^ cells/well) and HK-2 cells (1 × 10^4^ cells/well) were incubated in confocal glass bottom dishes. After 24 h, the medium was replaced with a medium containing the micelles or free drug and cultured for another 6 h. Then, the cells were treated with 4% paraformaldehyde for 20 min and stained with 2 mL DAPI solution for another 15 min. Finally, the cells were washed three times with 2 mL PBS solution and visualized with CLSM.

### In vitro cytotoxicity

The in vitro cytotoxicity and selectivity of the micelles were investigated by tumor cells (MDA-MB-231 cells) and normal cells (HK-2 cells) through the CCK-8 assay. In brief, MDA-MB-231 cells (1 × 10^4^ cells/well) and HK-2 cells (1 × 10^5^ cells/well) were cultured in 96-well plates for 24 h. Then, the cells were treated with the free drug mixture (the mixture of DOX and MTX) and the micelles with different concentrations. After 24 h, 10 μL CCK-8 solution was added to each well. The cells were incubated into the incubator for another 2 h, and then, the relative cell viability was calculated by measuring the absorbance at 450 nm with a microplate reader.

### Fluorescent probes staining

AO/EB staining was used to detect apoptosis of MDA-MB-231 cells. Briefly, MDA-MB-231 cells were planted in 12-well plates (5 × 10^5^ cells/well). After treatment, AO/EB mixture was added to each well for staining according to the protocol and then observed and photographed under a fluorescence microscope. DCFH-DA staining kit was used to detect the formation of reactive oxygen species (ROS). Briefly, the ROS was assayed with a DCFH-DA staining kit in accordance with the instructions provided by the manufacturer. MDA-MB-231 cells were seeded into six-well plate (2 × 10^5^ cells/well), after drug treatment, incubated in the dark for 30 min at 37 °C with 2 mL of working solution. Then, the cells were washed three times by PBS solution and visualized using the fluorescence microscope. The lysosomal escape capacity of the micelles was assessed by fluorescence microscope. MDA-MB-231 were plated in six-well plate (2 × 10^5^ cells/well). After drug treatment, LysoTracker Green staining was performed for 30 min, and the medium was aspirated and rinsed three times with ice-cold PBS solution, and then observed by fluorescence microscope.

### Western blotting

Briefly, MDA-MB-231 cells were plated in dishes overnight, and then, the medium was replaced with the free drug and the micelles solution for further incubation. After 24 h, the cells were washed three times with PBS solution, and total protein was isolated with RIPA lysis buffer. Subsequently, the protein was fractionated by sodium dodecyl sulfate polyacrylamide gel electrophoresis (SDS-PAGE) and transferred to the PVDF membrane. The PVDF membrane was sealed with BSA solution and then incubated with corresponding primary antibody overnight at 4 °C. Finally, the protein band was incubated with the secondary antibody, imaged by ECL and observed by the Bio Imaging system.

### Biological transmission electron microscopy

MDA-MB-231 cells were planted into a 6-well plate and cultured for 12 h at a density of 1 × 10^6^ cells/mL. Then, the cells were treated with free drug and the micelles and cultured for further 24 h. Subsequently, the culture medium was removed and added 2.5% glutaraldehyde to stabilize the cells. After 1 h, the cells were scraped off and centrifuged to remove the supernatant. Then slowly add 2.5% glutaraldehyde and store at 4 °C. The cells were prepared according to the procedure and observed using a biological transmission electron microscope.

### In vivo anti-tumor efficacy

Female Balb/c mice (18 ± 2 g, 5–6 weeks) were obtained from Beijing Vital River Laboratory Animal Technology Co., Ltd. The MDA-MB-231 tumor-bearing mice model was constructed by subcutaneous injection of MDA-MB-231 cells at a density of 2 × 10^6^ cells/mL into the axilla. After the tumor volume reached 100 mm^3^, the mice were randomly divided into three groups and treated with saline, free drug and the micelles on 0, 3, 6, 9, 12 and 15 days. The tumor volume and body weight were recorded every three days. The tumor volume was measured with a vernier caliper, and the volume (*V*, mm^3^) was calculated by the following formula: *V* = *A* × *B*^2^/2. (*A* represents the maximum diameter, and *B* represents the minimum diameter.)

### Biodistribution

The MDA-MB-231 tumor-bearing mice model was used to evaluate the biodistribution behavior of the drugs. After the tumor volume reached about 100mm^3^, the mice were intravenously injected with saline, the free drug and the micelles, respectively, through the tail vein at a dose equivalent to 5 mg/kg of DOX. At the scheduled time, the mice were killed, and the liver, heart, kidney, lung, spleen and tumor of the mice were taken to analyze the fluorescence distribution through the in vivo imaging system (excitation filter 475 nm, emission fluorescence 500–750 nm).

### Histological examination

At the end of the efficacy study in mice, the main organs (liver, heart, kidney, lung, spleen) and tumor tissues were collected for histopathological examination. All tissues were fixed with 4% paraformaldehyde and then dehydrated with gradient ethanol, embedded in paraffin, sectioned and baked. After deparaffinization and dehydration with xylene and absolute ethanol, all the slices were stained with hematoxylin and eosin and observed under a microscope.

### Statistical analysis

All results were represented as the mean ± standard deviation (SD), and statistical analysis was analyzed by Origin 8.5 software and SPSS 16.0. Statistical analysis of group differences was performed by Student’s t test. *p* < 0.05 was considered statistically significant (***p < 0.001, **p < 0.01 and *p < 0.05).

## Results and discussion

### Synthesis and characterization of the micelles

**Scheme 1:**
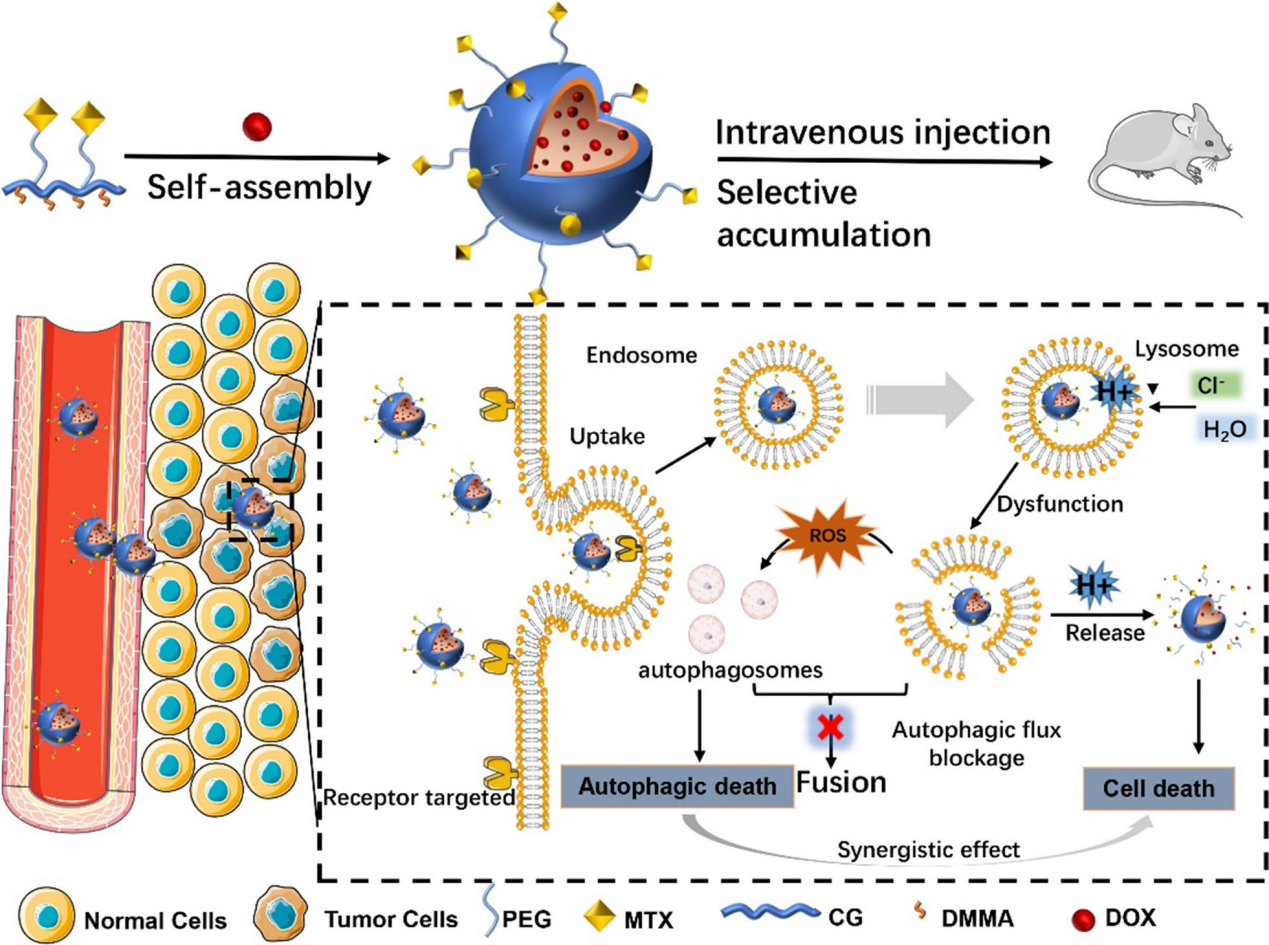
Schematic illustration of MTX-PEG-modified CG/DMMA polymeric micelles with autophagic flux interference and drug delivery

The detailed synthetic step of the micelles is presented in Scheme 1. First, MTX-PEG and CG synthesize CG-MTX-PEG under the action of the catalyst. Second, DMMA occupied the amino group on the CG-MTX-PEG under the catalysis of TEA. Finally, DOX was loaded with the electrostatic interaction of the carboxyl groups to form the micelles.

**Fig. 1:**
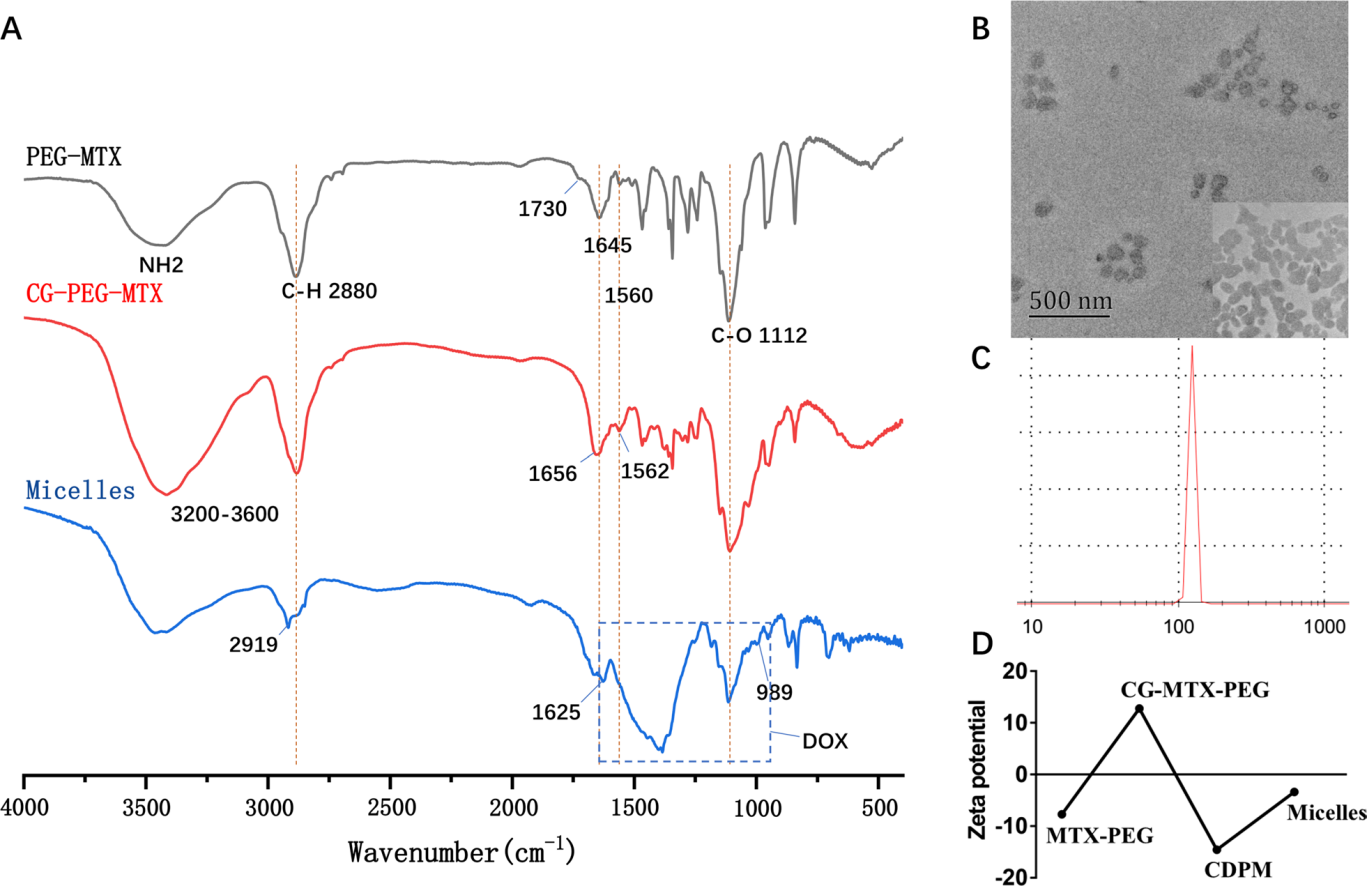
Synthesis and characterization of the micelles. **A** FT-IR spectra of MTX-PEG, CG-MTX-PEG and the micelles; **B** TEM images of the micelles; **C** representative size distribution of the micelles; and **D** Zeta potential changes in different steps

As revealed in the FT-IR spectrum (Fig. 1A), the stretching vibration of C–O and C–H in MTX-PEG appeared at 1112 cm^−1^ and 2880 cm^−1^, respectively, which are the feature absorption bands of PEG [32]. In addition, the characteristic absorption band of carboxyl group at 1730 cm^−1^ was also observed. After connected to the CG, the absorption band of NH_2_ and OH at 3200–3600 cm^−1^ was significantly enhanced. Signals at 1656 cm^−1^ and 1562 cm^−1^ are characteristic absorption bands of amide I and amide II, respectively, which indicated that CG and MTX-PEG were successfully connected through the amide bond [33]. The broad absorption band of the carboxyl group at 3300–2500 cm^−1^ and the absorption peak of –C=C– at 1625 cm^−1^ was observed in the micelles spectrum, which confirmed that DMMA was brought into the micelles system [34]. The absorption peaks 1255 cm^−1^, 1114 cm^−1^ and 989 cm^−1^ were identified to be related to DOX, which suggests that the DOX was successfully loaded into the micelles [35, 36].

The morphology of the micelles was observed by TEM. As shown in Fig. 1B, the micelles were uniformly dispersed with regular spherical shape. However, MTX-PEG, CG-MTX-PEG and CDPM have not presented a spherical structure (Additional file 1: Fig. S1), which proves that DOX successfully loaded into CDPM and forms nanospheres through self-assembly process. The size was monitored, and the results are shown in Fig. 1C. The micelles showed a narrow particle size distribution, and the mean particle diameter was 122 ± 3 nm. In addition, the micelles exhibited DOX and MTX loading capacity of ~ 6.5% and ~ 2.9% (weight ratio), respectively.

To detect the synthesis of the micelles, the Zeta potential of the product during the synthesis process was detected. As shown in Fig. 1D, Zeta potential exhibits significant changes in the synthesis process. After MTX-PEG connected with CG, the Zeta potential was transformed from − 7.72 to 12.8 mV, which may related to the natural cationic polysaccharide structure of CG. However, after the DMMA occupied the amino group, the potential of the CDPM becomes − 14.6 mV, and the potential of the system was turned to negative again. After the DOX was loaded, the micelles potential appears to − 3.4 mV. From MTX-PEG to the micelles, the Zeta potential changes in different steps, indicating that each step in the process was a successful modification. Besides, the slightly negative charge of the micelles can improve the protein resistance in the blood circulation and reduce the undesirable clearance of the reticuloendothelial system [37–39].

### In vitro drug release

The free drugs DOX and MTX were incorporated into the micelles by electrostatic interaction and covalent binding, respectively. Since pH value was considered to have important effect on the release of DOX from the micelles, the in vitro release behavior of the DOX was observed in pH 7.4 (physiological environment) and pH 5.4 (endosomal compartments). A variety of lysosomal proteases in tumor cells can cleave the amide linkage, which can promote the release of the MTX from the micelles to expose the anticancer effects. Therefore, the in vitro release behavior of MTX was carried out under PBS solution with or without the presence of crude protease.

**Fig. 2:**
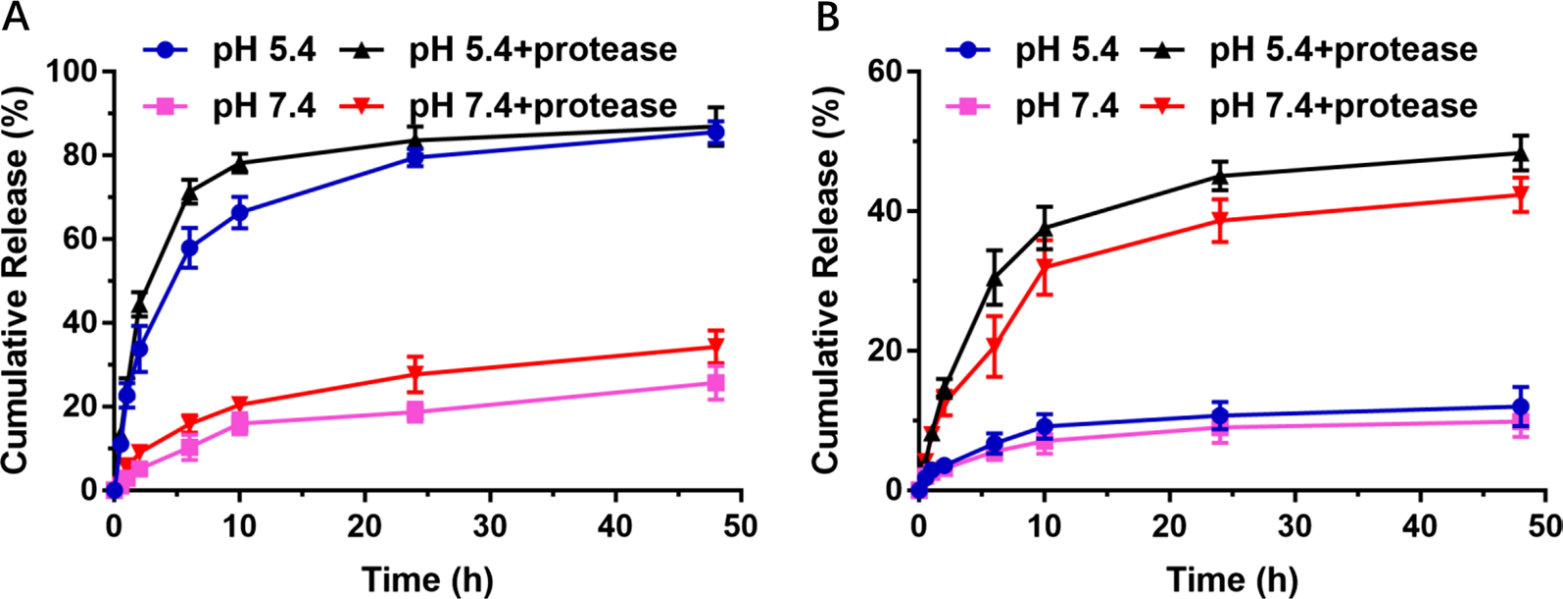
The drug release profiles of DOX (**A**) and MTX (**B**) from the micelles

As depicted in Fig. 2A, the release behavior of DOX was significantly regulated by the pH value. Specifically, at pH 7.4, only 25.7% of the DOX was released from the micelles, whereas the accumulative release amount increased to 85.6% at pH 5.4 after incubation for 48 h. Particularly, the release rate of DOX from micelles at pH 5.4 from the micelles was extremely high, particularly in the initial phase. The pH-dependent release profiles of DOX may be related to the decrease in ionization degree of carboxyl group under acidic conditions, which destroys the electrostatic interaction between DMMA and DOX. In addition, the accelerated release behavior of DOX under acidic conditions may be affected by its increased hydrophilicity under acidic conditions [40]. Such accurate and variable DOX release properties impart the results of low leakage behavior in normal physiological conditions and selective release capabilities in a tumor microenvironment.

However, the release behavior of MTX from the micelles was mainly promoted by protease. The cumulative release of MTX was significantly different in the presence or absence of protease in the same acidic environment. As shown in Fig. 2B, MTX accumulated release was 12.0% at pH 5.4, while the accumulated release reaches 48.4% with the presence of crude proteases over the same incubation for 48 h. Importantly, the release of MTX was not only affected by proteases, but also interfered with acidic environments. This result may be related to the stronger protease activity in acidic environment. Moreover, the cumulative release of MTX did not continue to increase after incubation for 24 h, which may be due to the decrease in enzyme activity over time. The release behavior of MTX after the amide bond ligation provides the micelles with selectivity to tumor tissues and low toxicity to normal tissues.

### Protein adsorption

Serum proteins in blood, which are mostly negatively charged, tend to bind to positively charged micelles by electrostatic interaction, leading to aggregation [41]. To demonstrate whether PEG and DMMA modifications can reduce nonspecific absorption, BSA was used as a biomolecules model to evaluate the absorption of MTX-PEG, CG-MTX-PEG, CDPM and the micelles, respectively. As shown in Additional file 1: Fig. S2, CG-MTX-PEG had the highest protein absorption, which was significantly reduced after DMMA modification, suggesting that charge reversal after DMMA decoration contributed to the reduction of nonspecific protein absorption. Compared with CG-MTX-PEG and CDPM, MTX-PEG and the micelles show lower absorption, which may be related to the inert structure of PEG. The inert structure of PEG can effectively reduce protein adsorption, especially when it was covered on the surface of the micelles, which can effectively reduce protein contact and improve the stability of the system [27, 42]. These results demonstrate that the negatively charged PEG-covered micelles can effectively reduce protein adsorption in blood circulation.

### Cellular uptake

**Fig. 3:**
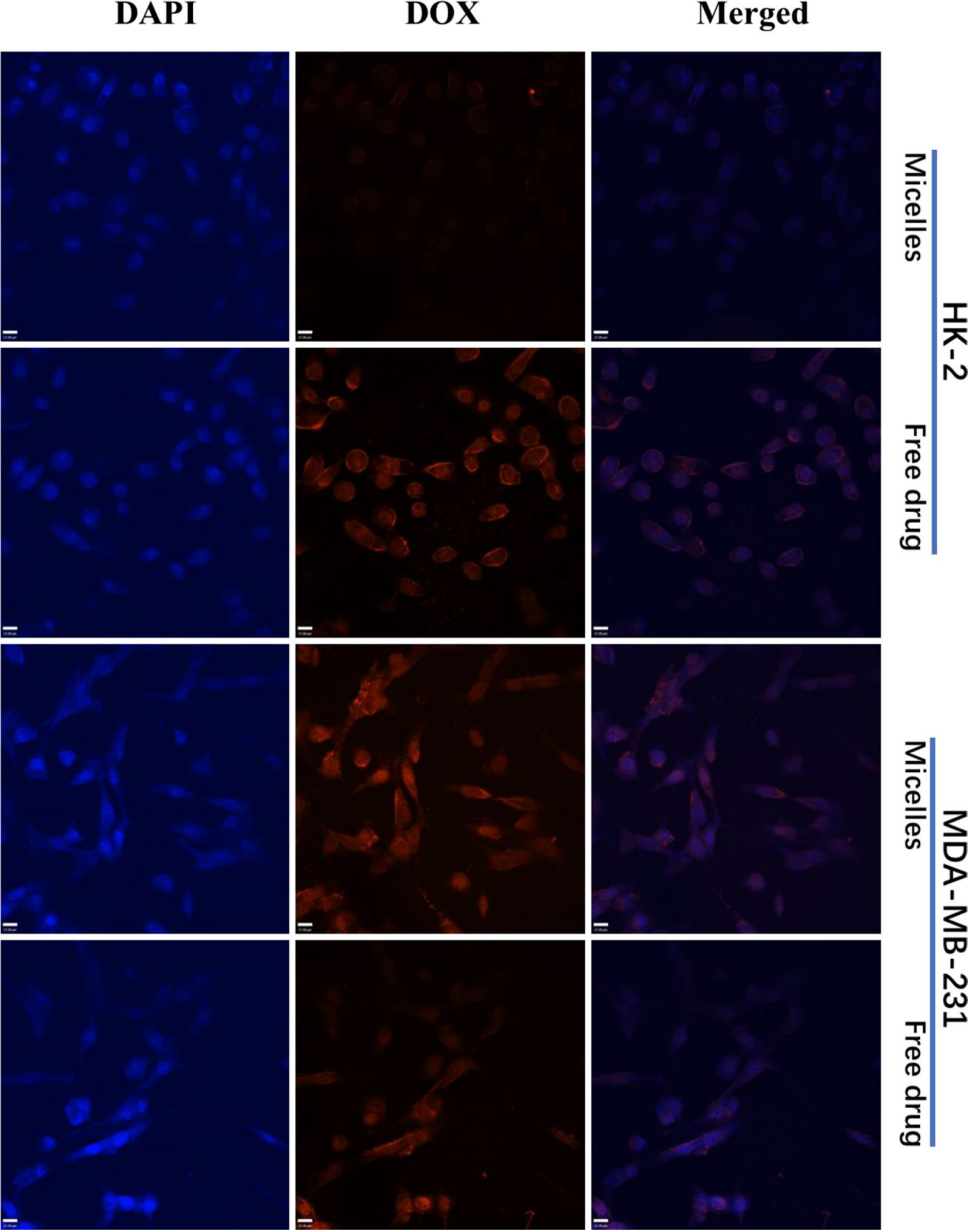
Confocal microscopic images of HK-2 cells and MDA-MB-231 cells after incubation with the micelles and free drug. The cell nucleus was stained with DAPI (blue), and DOX emits red fluorescence

To investigate whether the MTX-modified micelles can provide selectivity between tumor cells and normal cells, MDA-MB-231 and HK-2 cells were used to observe cellular uptake behavior of micelles. MDA-MB-231 and HK-2 cells were treated with free drug and the micelles for 6 h, respectively, and their cellular uptake behavior was visualized with CLSM. As depicted in Fig. 3, free drug shows similar results between normal cells and tumor cells. Red fluorescence (DOX) was dispersed in two cells, but the fluorescence of DOX in MDA-MB-231 cells was located in the nucleus overlayed in blue fluorescence (DAPI). However, red fluorescence exhibits significant differences between MDA-MB-231 and HK-2 cells treated by the micelles. In normal cells, only weak fluorescence can be observed, while tumor cells exhibit significantly reinforced red fluorescence in nucleus and cytoplasm. These phenomena may be attributed to different cellular uptake mechanisms.

DOX, as a small molecule chemotherapy, can pass the cell membrane by fast diffusion [40]. Therefore, the free drug lacks selectivity between normal cells and tumor cells. However, the micelles were mainly through the cell membrane via endocytosis pathway. MTX can specifically bind to folic acid receptors, while folic acid receptors are expressed in a variety of tumor cells including MDA-MB-231 cells [43]. These reasons determine the difference in cellular uptake of DOX loaded by micelles between MDA-MB-231 and HK-2 cells.

### In vitro cytotoxicity assays

**Fig. 4:**
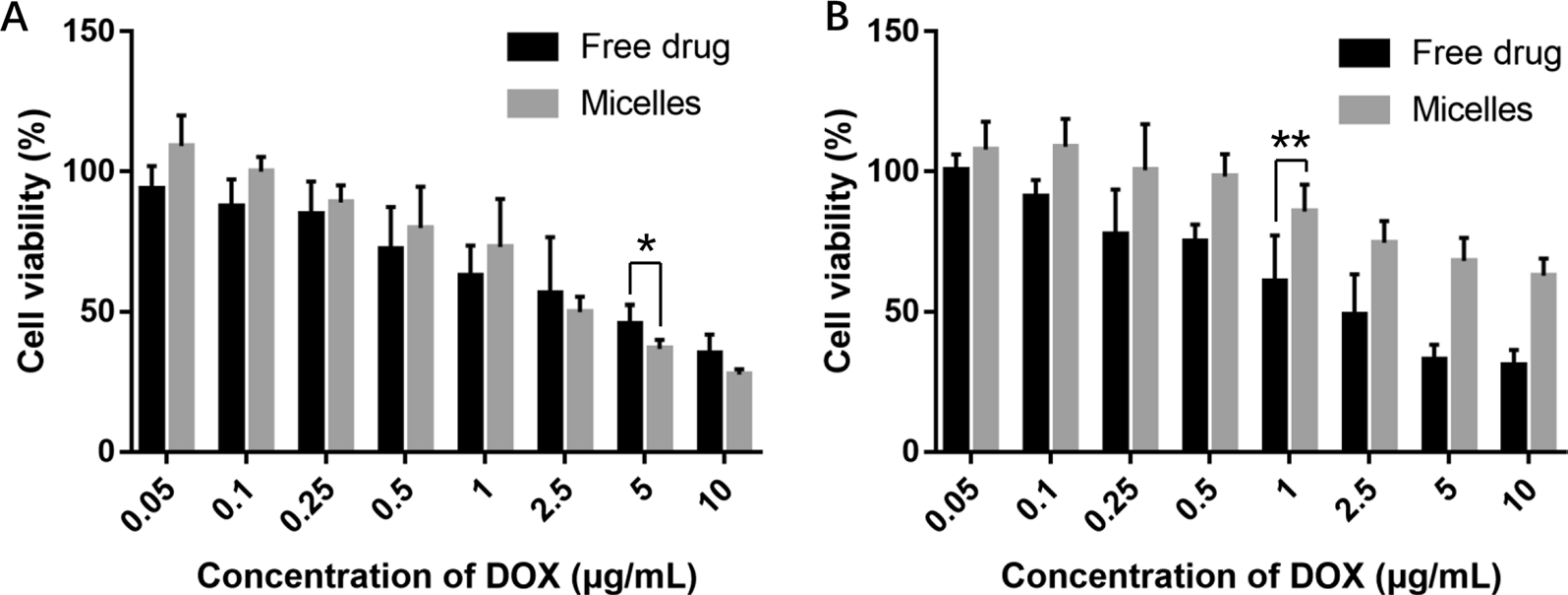
The cytotoxicity of the free drug and the micelles against MDA-MB-231 cells (**A**) and HK-2 cells (**B**). *p < 0.05, **p < 0.01 and ***p < 0.001

The in vitro cytotoxicity was evaluated in MDA-MB-231 and HK-2 cells at varying equivalent DOX concentrations using CCK-8 assay. As shown in Fig. 4A, in comparison with the free drug, the micelles showed similar concentration-dependent inhibition against MDA-MB-231 cell proliferation. By comparison, there was a significant difference in the therapeutic effect of the free drug and the micelles on HK-2 cells. As can be seen from Fig. 4B, with the increase in the concentration, the survival rate of cells treated by micelles declined slowly. When the concentration reached 10 μg/mL, the cell survival rate was still 62.8% after treated by the micelles, while only 30.9% in the free drug group. These results indicate that MTX-targeted micelles can not only significantly exert anti-proliferation effects on tumor cells, but also effectively reduce the cytotoxicity to normal cells.

### The effect on apoptosis and cell motility

To demonstrate the effect of micelles on apoptosis, AO/EB staining assay was used to detect apoptosis of MDA-MB-231 cells under free drug and micelle treatment. As shown in Additional file 1: Fig. S3, both micelles and free drugs showed higher apoptosis (red fluorescence) in MDA-MB-231 cells. In order to investigate whether the inhibitory effect of free drugs on the motility ability of MDA-MB-231 cells was affected after micelle loading, wound healing experiment was applied to study the inhibitory effect of free drugs and micelles on the motility of MDA-MB-231. As shown in Additional file 1: Fig. S4, the cells in the control group showed a high motility capacity (healing rates: 58.51%), while the healing rates in the free drug and micelle treatment groups were 13.73% and 17.37%, respectively. Combined with the above results of AO/EB staining and wound healing experiment, it was confirmed that the micelles still maintained the effect of free drugs to promote the apoptosis and inhibit the motility in tumor cells.

### The effect on oxidative stress

**Fig. 5:**
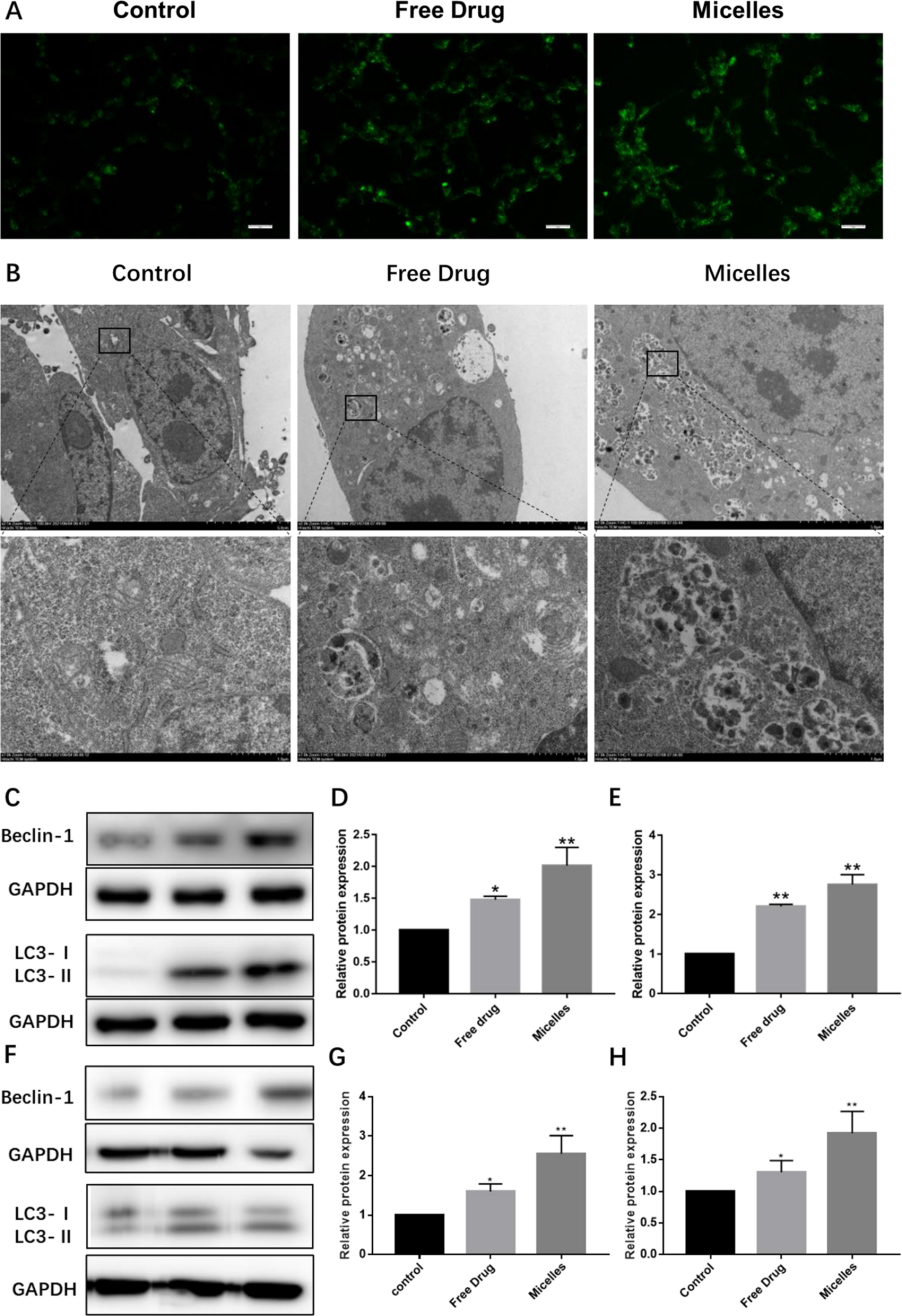
The effect of autophagic activation. **A** The micelles enhance oxidative stress in tumor cells. **B** The micelles-induced aggregation of autophagosomes; **C**–**E** Western blot of the Beclin-1 (**C**, **D**) and LC3-II (**C**, **E**) expressions in vitro; and (**F**–**H**) Western blot of the Beclin-1 (**F**, **G**) and LC3-II (**F**, **H**) expressions in vivo. *p < 0.05, **p < 0.01 and ***p < 0.001

Oxidative stress plays a role in the activation of apoptosis and autophagy, and the micelles-induced inhibition of tumor cells may utilize mechanisms associated with oxidative stress. To monitor the effect of the micelles on the oxidative stress in tumor cells, DCFH-DA was used to evaluate the level of oxidative stress in MDA-MB-231 cells. As depicted in Fig. 5A, compared to the control group, ROS levels were significantly increased in the micelles treatment group. The consistent results were found by flow cytometry. As shown in Additional file 1: Fig. S5, the micelles significantly increased ROS levels in MDA-MB-231 cells compared with the free drug and the control group. These results suggest that the micelles can induce oxidative stress in MDA-MB-231 cells, and the mechanism of oxidative stress induction may be related to DOX and CG, both of which have been verified to induce ROS accumulation [30, 44].

### The effect of autophagy activation

In addition, in case of oxidative stress, ROS levels were elevated, which positively contributes to autophagy, whether autophagy participated in the micelles mediated of cell death was further investigated [45]. Biological transmission electron microscope is the most convenient and intuitive way to observe autophagy. Therefore, this instrument was applied to observe the ultrastructure and morphology of MDA-MB-231 cells after treatment. As shown in Fig. 5B, in comparison with untreated controls, many autophagosomes, which are intracellular double-membraned vesicles characteristic of autophagy, were found in the free drug and the micelles-treated group. In addition, a large number of large autophagic vacuoles and small double/multimembrane vesicles occupied the major cellular space in the micelles-treated cells compared with free drug treatment. These results clearly indicated the occurrence of autophagy and the massive cellular autophagic structures might lead to cellular autophagy [45].

To further confirm the autophagy induction effect of the free drug and the micelles, the essential markers denoting the formation of autophagy were evaluated by western blotting. As an autophagy marker, the expression level of LC3-II indicates the extent of autophagosome formation. According to Fig. 5C, the incubation of MDA-MB-231 cells with the micelles significantly enhanced the ratio of LC3-II/LC3-I confirmed the effect of the micelles on autophagy activation. Quantitatively, the ratio of LC3-II/LC3-I after treatment of free drug and the micelles was 2.20 and 2.75, respectively (Fig. 5E). Additionally, the Beclin-1 level, an essential marker denoting the formation of autophagosome [30], was up-regulated by the treatment of the micelles as depicted in Fig. 5C. The results of semiquantitative analysis showed that the Beclin-1 ratio was 2.01 after micellar treatment, while the ratio of free drug treatment was 1.47 (Fig. 5D), indicating that the micelles effectively promoted the formation of autophagosomes, which was consistent with the results of Bio-TEM. Consistently, similar trends of Beclin-1 (Fig. 5F, G) and LC3-II/LC3-I (Fig. 5F, H) were detected in tumor tissue. The above results suggest that the micelles essentially affect the formation of autophagosomes.

### The blockade of autophagic flux

**Fig. 6:**
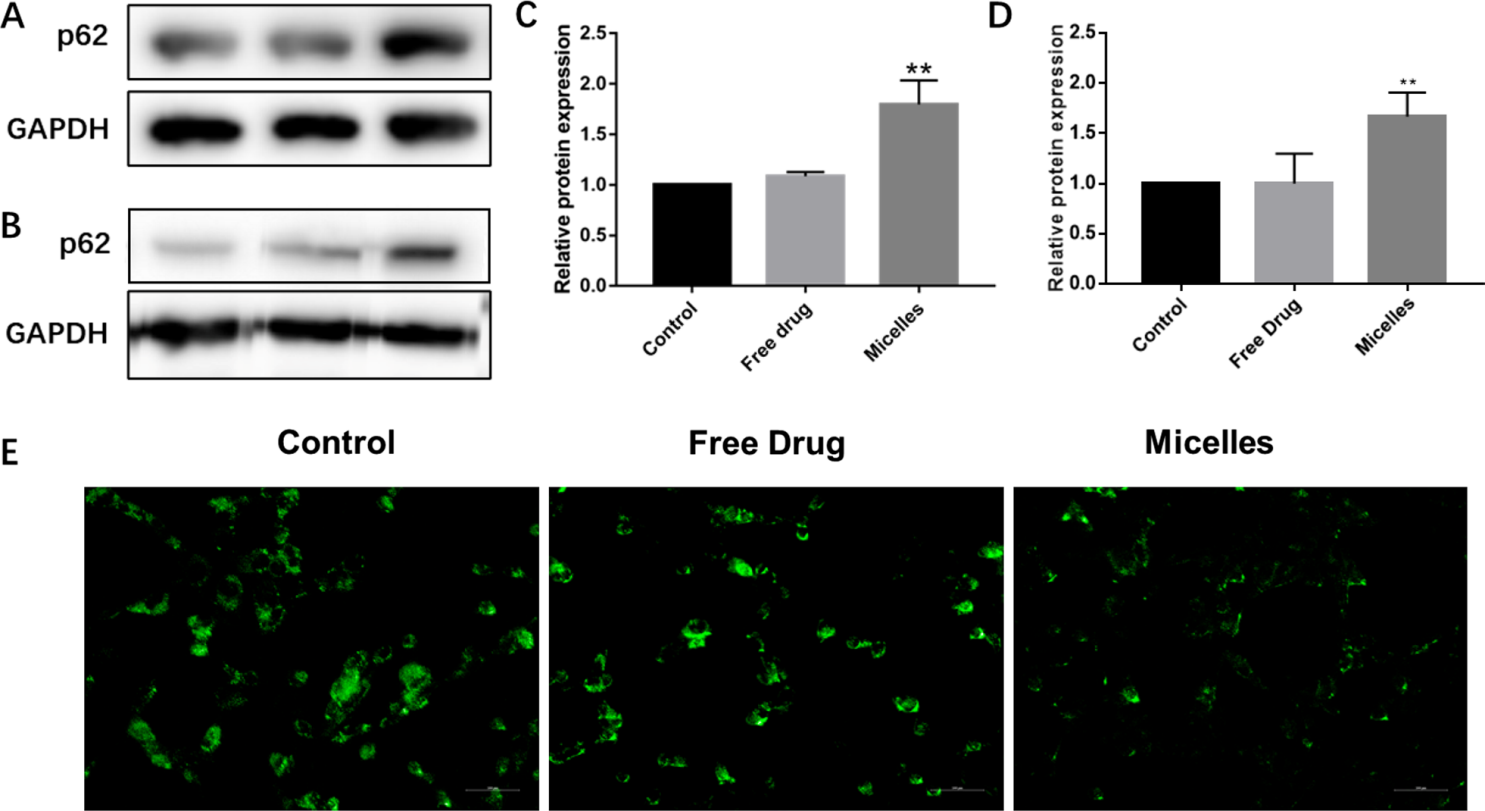
The blockade of autophagic flux. **A**–**D** Western blot of the p62 expressions in vitro (**A**, **C**) and in vivo (**B**, **D**); and **E** The fluorescence microscopy images of MDA-MB-231 cells. *p < 0.05, **p < 0.01 and ***p < 0.001

The final step of autophagy is the fusion with lysosome and degradation of the autophagosome. The normal function of lysosome ensures the smooth progress of autophagy, while the damage of lysosome will lead to the blockade of autophagic flux, which may further lead to the accumulation of autophagosome and induce autophagic death [46, 47]. p62, as an autophagy marker, is preferentially degraded during a normal complete autophagic process. Interestingly, autophagy is also thought to control the cellular levels of p62 protein through its lysosomal degradation. To observe the effect of the micelles on autophagy flux, the expression of p62 was measured in vitro (Fig. 6A, C) and in vivo (Fig. 6B, D). There was no significant difference in the expression of p62 after free drug treatment; instead, the increased expression of p62 appeared in vitro and in vivo after the micelles treatment, indicating that the micelles regulate autophagy flux.

In order to further analyze the effect of the micelles on autophagy flux, the stability of lysosomes was further analyzed. The optimal pH of most lysosomal enzymes is about 4.5. To test whether the micelles can affect environment of lysosomes, the cells were labeled with LysoTracker Green DND dye. LysoTracker Green DND, as an acid tropic dye, has a well-defined pH-dependent increase in fluorescence intensity and can be accumulated in acidic intracellular organelles. As shown in Fig. 6E, the intensity of green fluorescence in the micelles intervention was significantly lower than that in the free drug group and the control group, indicating that the micelles can promote lysosome alkalization and affect the stability of lysosomes.

Taken together, the micelles treatment can lead to accumulation of autophagosomes, and a large number of autophagic structures will eventually promote autophagic death. The mechanism of the micelles promoting autophagic death of cells may be related to CG and DMMA structures created defective lysosomes. The amide bond between CG and DMMA can break in the acidic lysosomal environment, and then, the primary amines of CG and the carboxyl group of DMMA capture the protons, resulting in a large number of chloride ions and water into lysosome, and eventually lysosome rupture [34, 48, 49]. Lysosome rupture led to hindered autophagosome fusion and slowed autophagy flux.

### In vivo anti-tumor efficiency

**Fig. 7:**
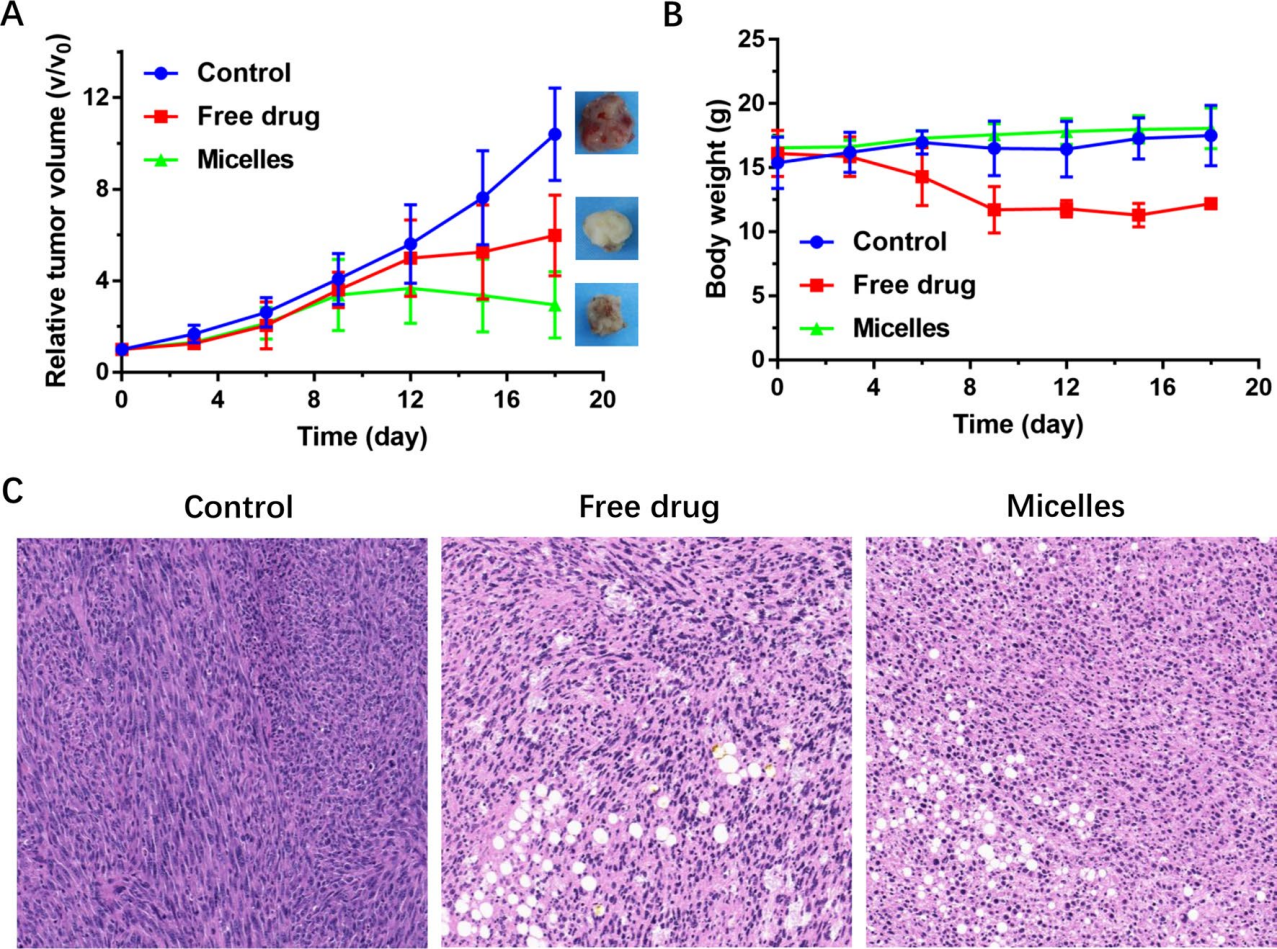
In vivo anti-tumor effect on MDA-MB-231 tumor-bearing mice. **A** Relative tumor volume (*V*/*V*_0_); **B** Body weight changes during administration; and **C** HE staining of tumor tissue

Considering the good tumor acid microenvironment responsiveness and the MTX active targeted effect of the micelles, the in vivo anti-tumor activity was evaluated in MDA-MB-231 tumor-bearing mice. The treatment was administered every three days. As can be seen from Fig. 7A, the tumor volume of mice in the control group increased rapidly, while both the free drug group and the micelles group showed tumor inhibition to varying degrees. After 9th day, tumor volume was significantly inhibited under micellar treatment compared with the other two groups, indicating a desirable tumor inhibition efficiency of the micelles.

The weight changes of mice during the treatment are shown in Fig. 7B. The weight of mice in the control group and the micelles group remained relatively stable throughout the treatment, while the mice treated with the free drugs showed abnormal weight loss, and even two mice died during treatment. These results indicated that the micelles not only maintain the therapeutic efficacy but also effectively mitigate the severe systemic toxicity of the free drugs.

To further assess anti-tumor efficacy, the tumors tissues were obtained after treatment for HE staining. As shown in Fig. 7C, it could be seen that there was a significant difference between the free drug group and the micelles group compared to the control group. Specifically, the histological feature appeared in the control group as dense tumor cells, while significant necrosis was observed in the free drug groups and the micelles groups. Further observation showed that the tumor cells treated with the free drugs and the micelles were mostly fusiform or irregular, and the tissue vacuolation was obvious. All these results indicated that the micelles possessed effective anti-tumor activity and negligible systemic toxicity.

### In vivo safety

**Fig. 8:**
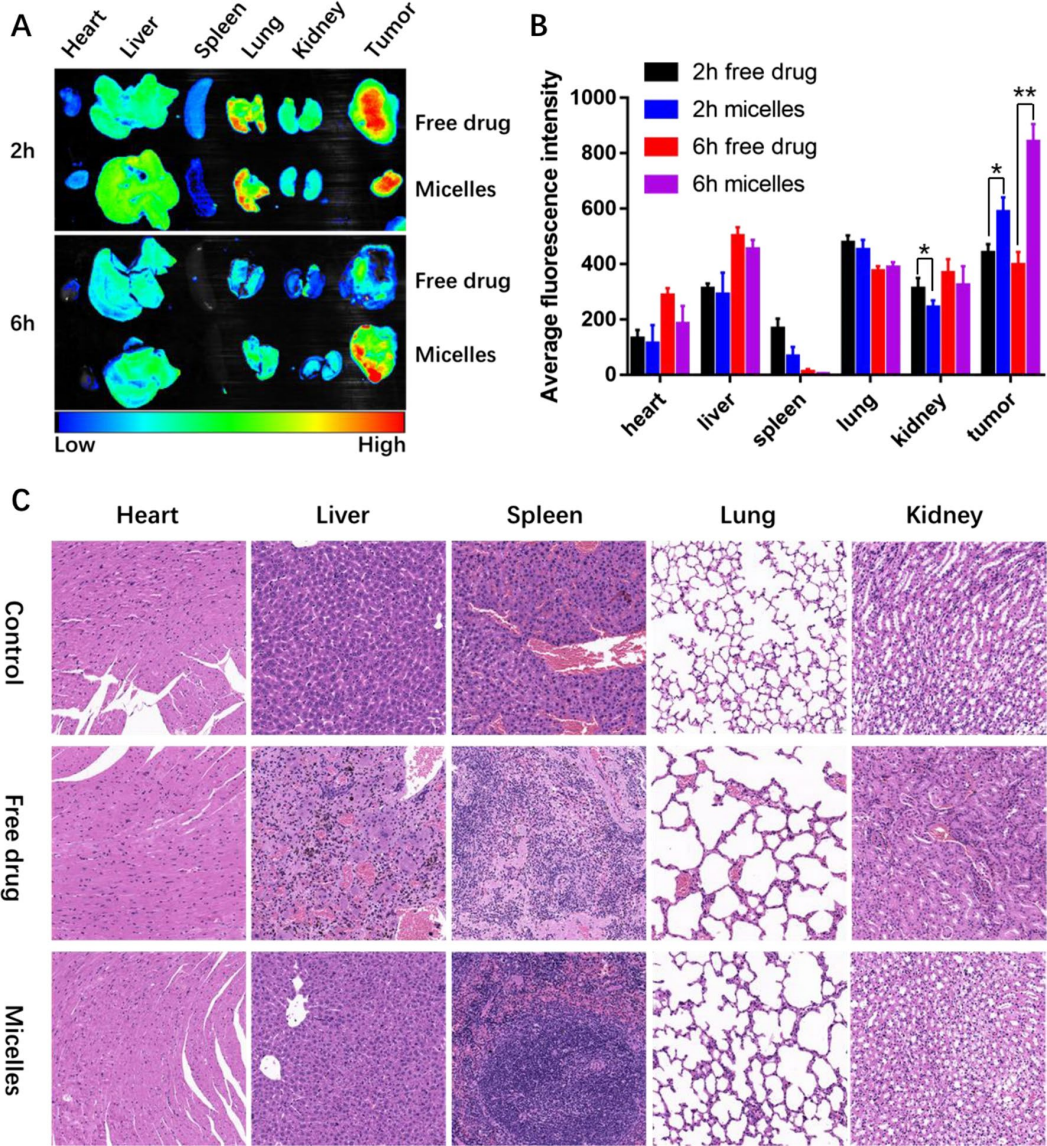
In vivo safety. **A** Ex vivo NIR images; **B** Average signals of the tumors and major organs after 2 and 6 h administration of the free drug and the micelles; and **C** Histopathological analysis of major organs after different treatments. *p < 0.05, **p < 0.01 and ***p < 0.001

The tissue biodistribution of a drug is an important aspect in assessing its effectiveness and potential organ toxicity as well as systemic side effects. In order to study the difference in the biological distribution of the micelles and free drug in MDA-MB-231 tumor-bearing mice, the ex vivo fluorescence imaging of MDA-MB-231 tumors and major organs isolated from BALB/c mice was carried out at 2 h and 6 h post-injection. The result is shown in Fig. 8A. In vivo biodistribution of the free drug and the micelles at 2 h after injection showed that the drug rapidly distributed in the major organs of mice and emitted strong fluorescence at the tumor site. However, the fluorescence intensity in the tumor treated by the micelles was significantly accumulated after 6 h as compared with that of the free drug group (Fig. 8B). This appreciable tumor-targeting ability of the micelles might result from appropriate size of the micelles and specific receptor affinity of the MTX.

To evaluate changes in systemic toxicity of the micelles compared with the free drug, major organs were collected and investigated by HE staining. As depicted in Fig. 8C, there were no evident necrosis, edema or inflammatory infiltration of major organs in the control group. However, major organs were damaged to varying degrees in the free drug group, especially in liver and kidney tissues. The liver tissue showed vacuolation, local necrosis and degeneration, and inflammatory infiltration and the renal tissue emerged glomerular hyperemia, swelling and necrosis. In addition, slight structural abnormalities and inflammatory infiltrates were observed in the heart, lung and spleen tissues. These phenomena indicate that free drugs have serious systemic toxicity. Compared with the free drug group, the damage of the main organs under the micelles-treated group was significantly improved. Only slight inflammatory infiltration was observed in liver and kidney tissues, and no significant damage was observed in other tissues compared with the control group, indicating that the micelles can effectively reduce systemic toxicity of the free drugs.

## Conclusion

In conclusion, the MTX-PEG-modified CG/DMMA polymeric micelles were designed and synthesized for targeted delivery of DOX to induce synergistic autophagic regulatory effects against TNBC. On the one hand, the MTX-PEG encapsulation improves stability and selectivity of the micelles, which effectively promotes specific aggregation of the micelles in tumor tissues, while reduces systemic toxicity and synergizes the anti-tumor efficiency of DOX. On the other hand, ROS under micellar therapy promotes the production of autophagosomes in tumor cells, while the lysosomal damage based on proton sponge effect blocks the autophagosome flux, leading to the accumulation of autophagosomes. Excessive accumulation of autophagosomes depletes normal cellular components and induces autophagic death of the stubborn tumor cells. Although the preliminary mechanism of autophagy has been verified, the deeper mechanism of micelles on autophagy flux has not been clearly explored. In addition, single cell lines and ectopic model were the limitations of this study.

## Supplementary Information

**Additional file 1: Figure S1**. TEM images of the PEG-MTX, CG-PEG-MTX and CDPM. **Figure S2**. BSA adsorption of PEG-MTX, CG-PEG-MTX, CDPM and the Micelles. *p < 0.05, **p < 0.01 and ***p < 0.001. **Figure S3**. AO/EB staining for apoptosis study on MDA-MB-231. The red fluorescence reflects the apoptotic state of cells. The green fluorescence reflects the normal state. **Figure S4**. Image of wound healing experiment of MDA-MB-231 cells. **Figure S5**. ROS levels were investigated by flow cytometry.

## Data Availability

All data generated or analyzed during this study are included in this published article and supplementary information files.
