# Supplementary material for: MTX-PEG-modified CG/DMMA polymeric micelles for targeted delivery of doxorubicin to induce synergistic autophagic death against triple-negative breast cancer
Source: Breast Cancer Res. 2023 Jan 12;25:3. doi: 10.1186/s13058-022-01599-9 (PMC9837947; doi:10.1186/s13058-022-01599-9)
Supplement: Supplementary file 1 — Additional file 1: Figure S1. TEM images of the PEG-MTX, CG-PEG-MTX and CDPM. Figure S2. BSA adsorption of PEG-MTX, CG-PEG-MTX, CDPM and the Micelles. *p < 0.05, **p < 0.01 and ***p < 0.001. Figure S3. AO/EB staining for apoptosis study on MDA-MB-231. The red fluorescence reflects the apoptotic state of cells. The green fluorescence reflects the normal state. Figure S4. Image of wound healing experiment of MDA-MB-231 cells. Figure S5. ROS levels were investigated by flow cytometry. [file 13058_2022_1599_MOESM1_ESM.docx]

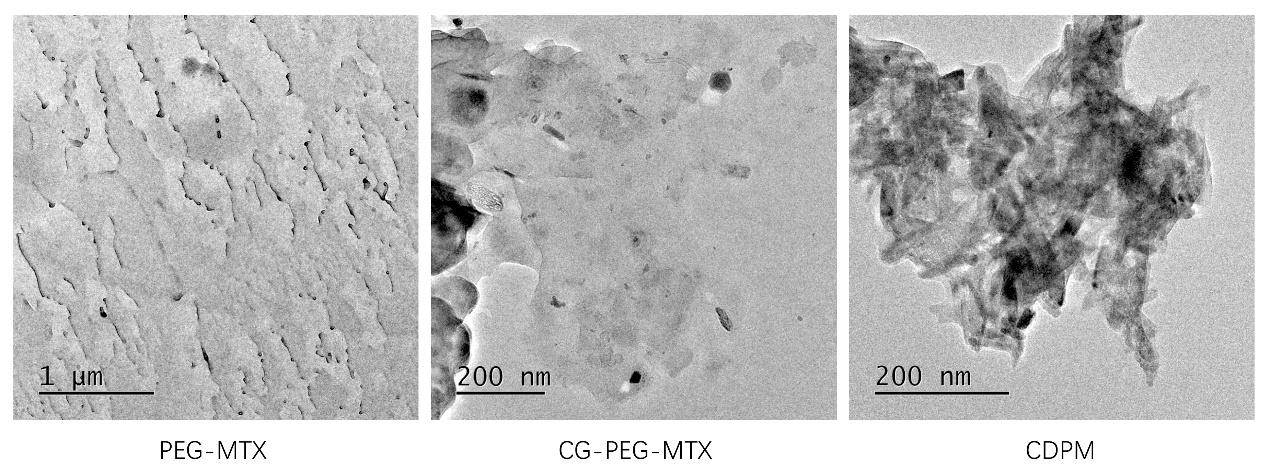


Additional file 1: Figure S1. TEM images of the PEG-MTX, CG-PEG-MTX and CDPM.


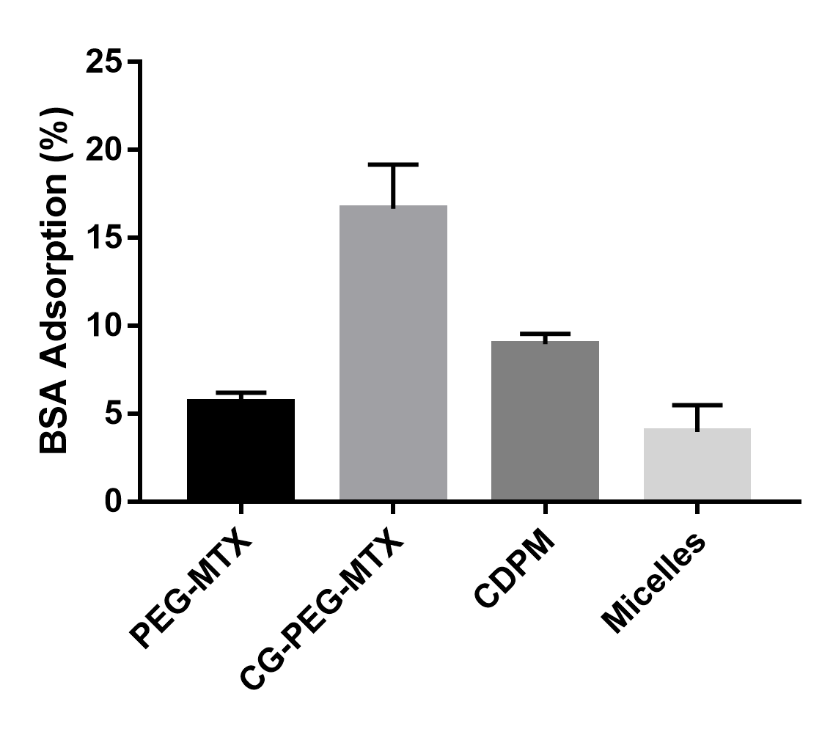


Additional file 1: Figure S2. BSA adsorption of PEG-MTX, CG-PEG-MTX, CDPM and the Micelles. **p* < 0.05, ***p* < 0.01 and ****p* < 0.001.


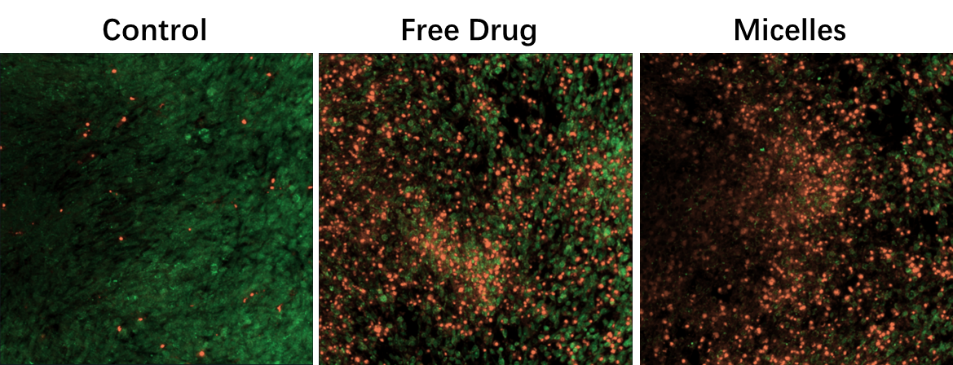


Additional file 1: Figure S3. AO/EB staining for apoptosis study on MDA-MB-231. The red fluorescence reflects the apoptotic state of cells. The green fluorescence reflects the normal state.


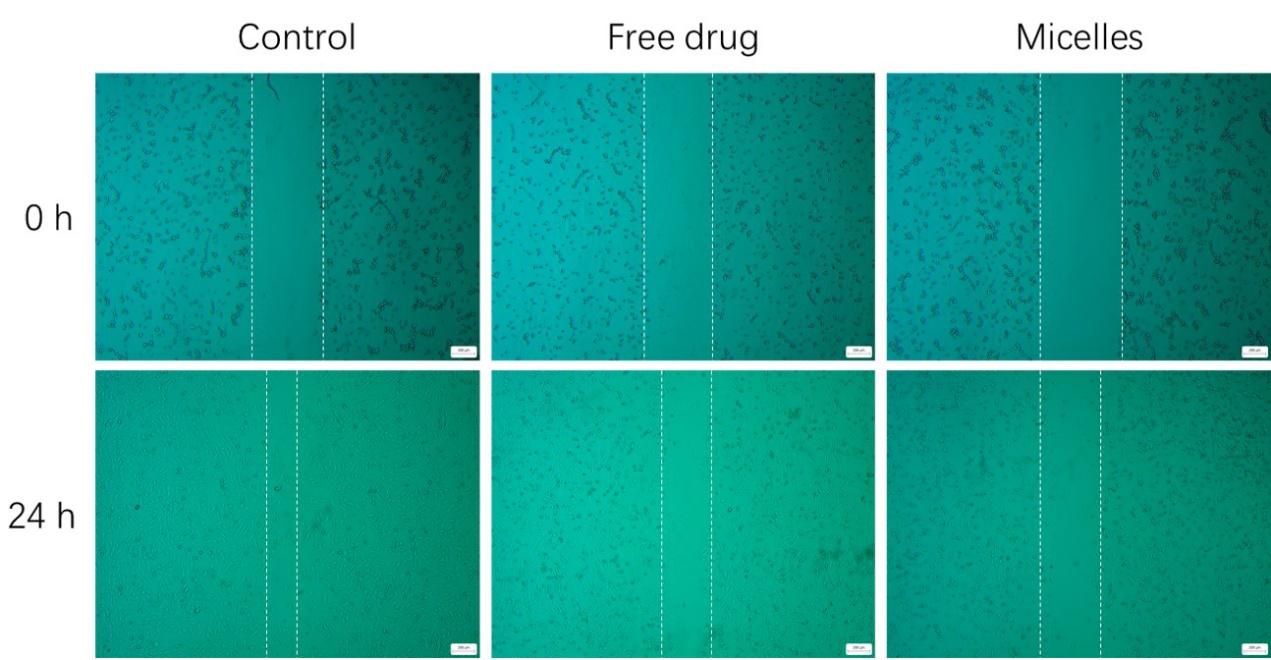


Additional file 1: Figure S4. Image of wound healing experiment of MDA-MB-231 cells.


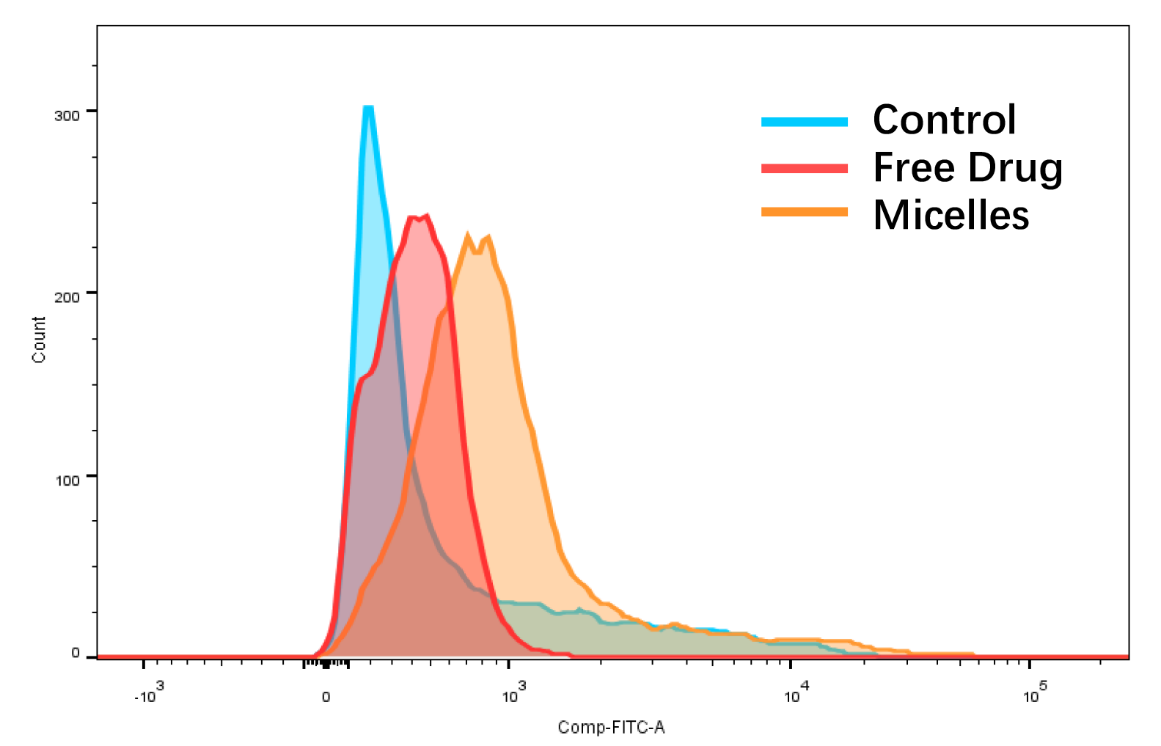


Additional file 1: Figure S5. ROS levels were investigated by flow cytometry.
